# Carcinogenicity of a Single Administration of N-Nitrosomethylurea: a Comparison Between New-born and 5-Week-Old Mice and Rats

**DOI:** 10.1038/bjc.1970.71

**Published:** 1970-09

**Authors:** B. Terracini, Maria C. Testa

## Abstract

**Images:**


					
588

CARCINOGENICITY OF A SINGLE ADMINISTRATION OF

N-NITROSOMETHYLUREA: A COMPARISON BETWEEN NEW-
BORN AND 5-WEEK-OLD MICE AND RATS

B. TERRACJNI* AND MARIA C. TESTA

From the Istituto Nazionate per lo Studio e la C(ura dei Tumori, 20133 Milano, Italy

Received for publication May 4, 1970

SUMMARY.-N-Nitrosomethylurea (NMUrea) was given as a single intra-
peritoneal injection either to newborn or to 5-week-old (C57BL x C3Hf)F, mice
and Wistar rats. Newborn mice were more susceptible than 5-week-old mice
to the development of lymphosarcomas, lung adenomas and hepatomas, whereas
newborn rats were more susceptible than their weaned counterparts to the
development of renal anaplastic tumours. Other tumours occurred with the
same frequency in newborn and mature animals. Tumours of the forestomach
in mice were more frequently found In animals treated at 5 weeks than in those
treated at birth. Since NMUrea persists for only a very short time and breaks
down spontaneously it seems that the paucity of enzymes related to immaturity
in newborns is not a major factor in determining the different susceptibility of
newborn animals to NMUrea carcinogenicity.

IN several experimental systems, newborn or suckling animals were found to
be more susceptible than mature animals to the effects of carcinogens. This
difference is not fully explained; among other hypotheses, it has been suggested
that the breakdown of chemical carcinogens is slower because of the enzymic
deficiency of young animals. Powerful carcinogens such as 7,12-dimethylbenz-
(a)anthracene, urethane and possibly N-nitrosodimethylamine persist unchanged
for longer periods in newborn than in adult laboratory animals (Domsky et al.,
1963; Mirvish et al., 1964; Terracini and Magee, 1964). N-nitrosomethylurea
(NMUrea) was felt to be a suitable compound for testing this hypothesis, since
in solution at pH around 7 it is unstable and is not likely to persist for more than
a few hours (Druckrey et al., 1967). NMUrea can produce tumours after a single
administration (Druckrey et al., 1964; Janisch et at., 1967; Kelly et al., 1968;
Leaver et al., 1969); its carcinogenicity in newborn mice and rats has been previ-
ously investigated (Kelly et al., 1968) but the available information does not
permit comparison of newborn and more mature animals as regards susceptibility
to tumour induction by NMUrea. Experiments on rats and different strains of
mice have been undertaken in this laboratory to elucidate this point. The
present work describes the effect of a single administration of NMJUrea to newborn
and 5-week-old (C57BL x C3Hf)Fl mice and Wistar rats. Whereas as a whole
newborns were more susceptible to tumour development than young adults,
inconsistencies between different target organs have been found. In addition,
several types of non-neoplastic lesions produced by NMUrea are described.

* Present address: Istituto di Anatomia Patologica, Universita di Torino, Torino, Italy.

CARCINOGENICITY OF N-NITROSOMETHYLUREA

MATERIAL AND METHODS

Hybrid (C57BL x C3Hf)F1 (BC3fF,) mice and Wistar rats from the colonies
of this laboratory were used. Animals of both species were fed a commercial
diet in pellets (Mangime Valleolona, Castellanza, Varese) and tap water ad libitum.
Animals were treated within 24 hours after birth or at 35 days. Recrystallized
NMUrea was obtained through the generosity of Mr. P. F. Swann, Courtauld
Institute of Biochemistry, Middlesex Hospital Medical School, London. It was
dissolved in saline immediately before use at a concentration of 0.1%. With the
exception of one experiment, injections were given intraperitoneally at a standard
dose of 50 ,ug./g., because preliminary experiments in newborn mice had demon-
strated that 100 ,ug./g. produced a high early mortality. One additional experi-
ment in rats was carried out with the purpose of directly exposing the thymus to
the carcinogen: newborns from 4 litters received an intrathoracic injection of 50 or
100 ,ug. NMUrea (i.e. in the order of 8-16 ,ug./g.) as a 0.5 % solution in saline. This
was done under ether anaesthesia and through opening in the chest in the animals
of one litter; rats of the other three litters received the solution of NMUrea as an
injection through the thoracic wall.

Rats and mice were weaned at 3-4 weeks of age and separated according to
sex. They were subsequently controlled daily and weighed at weekly or fort-
nightly intervals. The animals were allowed to die naturally or were killed with
ether if obviously sick: all survivors were killed at 60-64 weeks of age.

A control group of BC3fF1 mice was given only saline at birth. The pathology
of a control group of 95 Wistar rats has already been reported (Della Porta et al.,
1968). During the performance of the present study the weight and the survival
rate of the experimental rats were compared to that of an untreated group
assembled at about the same time, which was over 2 years of age at the end of 1969.

Complete autopsies were performed on all animals, including opening of the
spinal cord in rats. Histological sections were routinely prepared from the
thymus, liver, kidneys and spleen, with the exception of a few animals highly
decomposed. Endocrine organs were also examined in most rats. In addition,
all organs grossly damaged were examined histologically. Specimens were
fixed in Bouin and stained with haematoxylin-eosin: at least one coronary section
from each kidney was routinely examined.

RESULTS

Survival rates are presented in Table I for both mice and rats receiving NMUrea
i.p. and for control animals. Eleven of 95 mice treated at birth died before
weaning compared with 3 of 34 controls. Early deaths did not occur in mice
and rats treated at 5 weeks of age.

A consistent observation among animals treated at birth was in irreversible
impairment of body growth. Fig. 1 and Table II show the pattern of body growth
in mice and rats respectively. Growth depression was also observed, although to
a much lesser extent, among mice treated at 5 weeks of age. All mice and rats
treated at birth were smaller than the controls, regardless of whether they
developed tumours or not.

Tables III and IV show the incidences of tumours observed in mice and rats
respectively. Both tables indicate a high carcinogenic activity of NMUrea with
the production of a broad spectrum of tumours. Only 7 % of mice treated at

589

B. TERRACINI AND MARIA C. TESTA

TABLE I.-Survival Rates in BC3fF, Mice and Wistar Rats Treated with

NMUrea 50 ,ug./g. i.p. once at < 24 hours or at 5 Weeks of Age

Survivors at weeks of age

Groups
BC3fFj mice

Controls

NMUrea at birth

NMUrea at 5 weeks

Wistar rats

Controls

NMUrea at birth

NMUrea at 5 weeks

5    10    20    30     40    50    60

&
6'
6'
Is
Y3
6'

11
20
45
39
45
35

18
18
14
18
12

8

11
20
45
38
45
35

18
18
14
18
12

8

11
20
25
22
40
34

18
17
13
18
12

7

11
20
20
16
32
29

18
17

8
17
10

6

11
20
19
14
24
25

18
16
6
8
7
5

11
20
18
14
20
24

18
16
4
3
5
4

11
20
17
11
18
21

18
16
2
1
4
4

10    20    30    40    50   60

~~~~~~~~~~~~~~~............. ......... .... . .. .. ..... .. .. .

,f

10    20    30    40     50    60

WEEKS OF AGE

FIG. 1.-Body growth in BC3fF, mice given NMUrea 50 ,ug./g. as a single injection at 1 or 35

days of age.

.............. .Controls

?  -   - NMUrea at 1 day

NMUrea at 35 days

TABLE II.-Weight (in grams) of Wistar Rats given NMUrea 50 ,ug./g. as a Single

Injection at 1 or 35 Days of Age.    Number of Rats in Brackets

Age in weeks

Treatment
Controls

NMUrea at 1 day

NMUrea at 35 days

Sex

Y

6'
6'

6'

7

140+2 (18)
170+6 (18)
64+2 (14)
77+5 (18)
130+3 (12)
159+2 (8)

13

227?17 (18)
305+11 (18)
116?19 (14)
163+11 (18)
204+4 (12)
302+6 (8)

18

233+2 (18)
350+10 (18)
129+3 (13)
194+12 (18)
232+4 (12)
357+10 (7)

birth and 21% of those treated at 5 weeks were tumour-free, compared to 97%
among the controls. In rats treated either at 1 day or at 5 weeks, only 3 of 52
had no tumours at death. Multiple tumours in the same animal were commonly
found. With the exception of mice with lymphosarcomas and rats with large

35.
'I 30
< 25

20

z 20-

I- 15

I

o- 10*
3 5

590

CARCINOGENICITY OF N-NITROSOMETHYLUREA

0

0-0 ) c  l-

_     4 -D

oq      w~~~a

o I I :O

I  I  I  ' s

I I  e:c:  I a *

0-S  I m o  00 I

4OU eCO0
00    Ci0     00o

?I I m- 010 0

0q
o I I Ceb <zo ecz 4;O

o.0       o1      Co X

o*~-

Co               4 0

Co       +0               W

&         I

0 B               c

L -

591

0

0
C)
Co

0
fv

C)
4

oq

0
Co

0

t40
c-

0
0

0
40

;.4
C-

0
(1

{
I

P-

o

0

Co

0

r40

12

,
4D

~0
P-
Us

Co
0
o
Cs
40

*-

0

0
cCl

40
C3

0n

0

-4

0~

C)

0 2  I

01

P4

o

.

*

c)
f-

* 40

.40
ZZ;b
CO
C)
0s

IO

H *

I

m
C2

0

0

C3)
Ca

0.

0
m

OD
Ce
4
O-

4

I
c

B. TERRACINI AND MARIA C. TESTA

- *
to

0 O

Co

CO

*y

E- *

f

-'   t'4   I

a 0

(11

0
lS

m

p 0

CB

I d

" D

'P
03

{

I

1-

0S 0

4a d
2s

ww

d   0
1-

- 9t

4---

-o         0

0
o> O _ J

U,l

^- -,

____O

et  Ci C  .

0a

._q

'4-4

0

2

4,
0

o
X0

'4

*?

0

.2

0

0

;  a

~0  -0 t

00

.F  -

cs ce o

-- --oo--

'I o  r

0 -
- CA

c*'*o* o

4-U1

0 1               0

4'4           '

592

CARCINOGENICITY OF N-NITROSOMETHYLUREA

anaplastic tumours of the kidneys, it was difficult to establish which tumour was
causally related to death or sickness.

Lympho8arcomas.-Thymic lymphosarcomas and generalized lymphosarcomas
with prominent involvement of the thymus were the tumours most commonly
found in mice. There were great variations in the degree of involvement of organs
other than the thymus. Among mice treated at birth, the incidence of lympho-
sarcomas in both sexes ranged between 50 and 60% with an average age at death
of 17-18 weeks. The incidence of lymphosarcomas in mice treated at 5 weeks
of age was 46% in females and 31% in males, with an average age at death of
29 weeks in both sexes.

Lymphosarcomas were found in one rat treated at birth and killed at 38 weeks
of age, and in 3 rats observed at 16, 21 and 28 weeks of age among those treated
at 5 weeks. With one exception in which the thymus was spared, they were
generalized lymphosarcomas of probable thymic origin. No tumours of the
lymphatic organs were seen among rats injected NMUrea intrathoracically. In
95 untreated rats of this strain, one generalized lymphosarcoma occurred in a
rat dying at 17 weeks of age; another rat aged 113 weeks had hepatic and splenic
lymphoma.

Tumours and non-neoplastic lesions of the kidneys.-Three mice of each sex
among those treated at birth and 2 males treated at 5 weeks had cystic-papillary
or trabecular, non-invasive renal adenomas. Only one of the tumours was greater
than a few mm. in diameter and showed atypicalities, with no obvious invasion.
No renal tumours were found in the control group. In addition, 27 mice of either
sex treated at birth (including 4 with renal adenomas), 8 treated at 5 weeks of age
and 2 control males showed single or multiple " hyperplastic tubules " in the renal
cortex (Terracini et al., 1966) (Fig. 2). Finally, among mice treated at birth, but
not among those treated at 5 weeks or in the controls glomeruli with cell loss and
fibrosis, occasionally with involvement of the Bowman's capsule, were seen (Fig. 3).

Renal tumours were the most commonly observed neoplasms among rats treated
either at birth or at 5 weeks of age. They were bilateral in 11 rats treated
at birth and in 1 treated at 5 weeks. Two different types of tumours were found,
anaplastic or interstitial and tubular (Magee and Barnes, 1962; Riopelle and
Jasmin, 1969). The incidence of renal tumours among survivors at 20 weeks of
age was 74% in rats treated at birth and 37% in those treated at 5 weeks. Age
at death of rats with renal anaplastic tumours was roughly similar in both groups.
Only 1 anaplastic tumour was seen among 95 untreated rats from the same
colony.

Six rats with renal tumours of tubular origin were observed throughout the
present series. They were all less than 0*3 cm. in diameter and histologically
appeared as solid or papillary adenomas; some contained areas of necrosis but no
invasion or other signs of malignancy. In addition, 4 rats treated at birth had at
least 1 hyperplastic tubule. Among 95 untreated animals, one renal adenoma
and 2 adenocarcinomas were observed at an average age of more than 100 weeks.

Of the 32 animals of both sexes given NMUrea intrathoracically, 13 developed
a total of 17 anaplastic and 2 tubular tumours.

Lung adenomas.-They occurred only in experimental mice surviving the period
of high mortality due to lymphosarcoma. Animals treated at birth were more
susceptible than those treated at 5 weeks. With the exception of 4 animals of
either sex in the former group and 3 in the latter one, lung adenomas were found

51

593

B. TERRACINI AND MARIA C. TESTA

in mice killed at the end of the experiment. Considering survivors at 40 weeks,
incidences were 81O% and 38% among those treated at 1 and 35 days respectively.
Lung adenomas were usually multiple but only rarely did they largely replace
the lung parenchyma.

Hepatomas.-This type of tumour was also seen only in mice. Data contained
in Table III indicate a much higher susceptibility of males than females and a
sharp loss of susceptibility at 5 weeks of age. Hepatomas were more than 0-8 cm.
in diameter, on section they showed a trabecular pattern and compressed the
surrounding parenchyma without invasion. No hyperplastic nodules or other
lesions often associated with hepatocarcinogenesis were observed. Lung
metastases were not seen.

Tumours of the forestomach.-The relation between age at treatment and
subsequent tumour development appeared to be different in mice and rats.
Tumour incidence was higher in mice treated at 5 weeks than in those treated at
birth, whereas only rats treated when newborn developed this type of tumour.
In mice, with the exception of a male treated at 5 weeks and dying with a squamous
cell carcinoma at 54 weeks, all stomach tumours were papillomas found in animals
killed at the end of the experiment. The 7 tumours of the forestomach in rats
were papillomas observed in animals dying from other causes between the 33rd
and 50th week of life. In addition, 1 papilloma of the forestomach was found
among the rats given NMUrea intrathoracically. Four rats in the control group,
aged more than 100 weeks, each had a papilloma of the forestomach.

Tumours of the intestine.-The only intestinal tumour in a mouse was a sarcoma
in a male treated at birth. A total of 7 rats with intestinal adenocarcinomas were
found: 1 tumour was located in the duodenum, 5 in the small intestine and 1 in
the colon. An animal with intestinal adenocarcinoma also had a carcinoid of the
caecum. Two borderline lesions, possibly non-invasive adenocarcinomas, were
also seen, 1 of which was in a rat with an adenocarcinoma. Another adenocarcin-
oma of the intestine was found in 1 of the rats given NMUrea in the thorax. Two
adenomatous polyps were found in the control group.

Mammary tumours.-The only mammary tumour in a mouse was found in a
female treated at 5 weeks and dying at 42 weeks of age. In rats, 4 mammary
tumours were found among females treated at birth (all were palpable before the
30th week of age) and 3 among those treated at 5 weeks of age (all of which were
palpable after the 50th week of age). Two of the 17 females treated intrathora-
cically developed mammary tumours. Among the controls, 15 of 48 females
developed mammary tumours, the earliest being palpable at the 86th week of age.

EXPLANATION OF PLATES

FIG. 2. Female mouse given NMUrea at birth and killed at 30 weeks. Hyperplastic tubule

in the kidney. H. & E. x 420.

FIG. 3. Female mouse given NMUrea at birth and killed at 46 weeks. Renal glomerulus

showing thickening of Bowman's capsule and some increase of the mesangium. H. & E.
x 510.

FIG. 4. Male mouse given NMUrea at birth and killed at 22 weeks. Cutaneous cyst contain-

ing keratin. H. & E. x 90.

FIG. 5.-Same lesion as in Fig. 4. The cyst is lined by flat epithelial cells. H. & E. x 420.
FIG. 6.-Male rat given NMUrea at birth and killed at 44 weeks. Testicle showing atrophy

of the germinal epithelium and hyperplasia of the interstitial cells. H. & E. x 70.

FIG. 7. Male rat given NMUrea at birth and dying at 43 weeks. Intracranial neurinoma.

H. & E. x 420.

594

BRITISH JOURNAL OF CANCER.

2

3

Terracini and Testa

VOl. XXIV, NO. 3.

BRITISH JOURNAL OF CANCER.

4

5

Torracini and Testa

VOl. XXIV, NO. 3.

BRITISH JOURNAL OF CANCER.

6

*' .i.,..:.

A  A3

7

Terracini and Testa

VOl. XXIV, NO. 3.

CARCINOGENICITY OF N-NITROSOMETHYLUREA

Non-neoplastic lesions in mice.-The occurrence of hyperplastic tubules and
hyaline glomeruli in the kidney has already been mentioned. In addition, at
autopsy, in 29 mice treated at birth, but in none of those treated at 5 weeks or in
the control group, there were multiple dark spots in the inner side of the skin;
they were up to 02 cm. in diameter and histologically appeared as cysts contain-
ing keratin, lined by flat cells, associated with groups of melanocytes and occas-
ionally with small foreign body lesions. The earliest change of this type was
seen in a mouse dying at 15 weeks of age (Fig. 4 and 5). A common finding in
female mice given NMUrea either at birth or at 5 weeks of age and killed at the
end of the experiment was a dilation of the uterine horns associated with cystic
hyperplasia of the endometrium.

Non-neoplastic lesions in rats.-Among rats treated at birth, the incisor teeth
of several animals were irregular and very long and had to be cut several times.
This was unlikely to be a major cause of stunted growth since there were no
differences in body growth among animals with normal and abnormal teeth.

Another common finding in male rats treated with NMUrea at birth were
small testes. On section, the germinal epithelium appeared atrophic (Fig. 6) and
very few or no spermatozoa were present in the epididymis. A common finding
in these testes was hyperplasia of the interstitial cells, which in 2 rats appeared
as large areas classifiable as interstitial cell tumours (Table IV). Endometrial
changes were seen only in a female treated at birth. Ovarian cysts up to 1 cm.
in diameter were observed in 5 instances. No consistent changes were found in
the pituitary, adrenals and thyroid.

DISCUSSION

Although a single administration of 50 ,tg./g. NMUrea proved to be highly
carcinogenic to both species investigated, the type and location of tumours was
different in mice and rats. This confirms a previous observation on the effects
of NMUrea in newborn animals (Kelly et al., 1968) and at present can be explained
only on a speculative basis. Intrathoracic administration of carcinogens is
known to enhance the occurrence of thymic lymphosarcomas in mice (Chieco-
Bianchi et al., 1965; Doell et al., 1967) and this could be related to a higher amount
of the carcinogen reaching the target organ; in the present study, however, rats
given NMUrea intrathoracically failed to develop thymic lymphosarcomas.

In both mice and rats there were differences in tumour incidence between
animals treated at birth and later in life. The present study confirms that lympho-
sarcomas, hepatomas and lung adenomas in mice as well as renal anaplastic tumours
in rats are more easily induced in infant than in mature animals (Toth, 1968;
Della Porta and Terracini, 1969). The earlier occurrence of mammary tumours
in rats given NMUrea indicates a similar trend. However, in mice, stomach
tumours occurred more frequently in animals treated at 5 weeks of age. The
tumours at different sites indicated in the footnotes of Tables III and IV were
probably related to the treatment and their incidence was not significantly
different in animals treated at birth or later in life.

Since NMUrea breakdown is rapid and may not require an enzyme (Leaver et al.,
1969) it seems that factors other than the degree of maturation of enzyme produc-
tion are related to the difference in susceptibility among newborns and young
adults. Thus, the " organotropism " (Druckrey et al., 1967) of NMUrea is different
in mice and rats and is influenced by the age at treatment. Present knowledge is

595

B. TERRACINI AND MARIA C. TESTA

insufficient to establish whether species- and age-related differences are the conse-
quence of a different rate of absorption, a different distribution of the carcinogen
or the different functional state of some organs in newborn animals.

NMUrea ranks among the most potent leukaemogenic chemicals in mice as
single doses of 30 ,ug./g. or higher have produced incidences of lymphosarcomas of
40%0 or more in all strains so far investigated, i.e. XVII (Graffi and Hoffmann,
1966), outbred Swiss (Terracini and Stramignoni, 1967), inbred Swiss (Frei, 1969),
NIH general purpose (Kelly et al., 1968) and BC3fF1 (in the present study). The
order of magnitude of the effective doses and the percentage of mice developing
lymphosarcomas are comparable to those observed following administration of
7, 12-dimethylbenz(a)anthracene (Toth et al., 1963); a single administration of
urethane to newborn mice was equally effective only when given at a dose of
1 mg./g. and in Swiss mice (De Benedictis et al., 1964) whereas in C3Hf, C3H,
BC3F1 and C57BL mice a longer exposure to urethane was required to produce
lymphosarcomas (Della Porta et al., 1967). In the present study, when 35-day-old
mice were used, lymphosarcomas were induced, but their incidence was somewhat
lower and the latent period (measured as age at death) was longer than in mice
treated at birth. In the case of urethane in Swiss mice, susceptibility to the leukae-
mogenic effect of 1 mg./g. was found to decrease significantly between 1 and 40 days
of age (IDe Benedictis et al., 1964).

A different situation is created by lung tumours: the decreased ability of
NMUrea to induce lung adenomas in mice aged 35 days contrasts with the observa-
tion that in experiments lasting at least 30 weeks, following single doses of DMBA,
nitroquinoline oxide and urethane, the incidence of lung adenomas approached
100% in animals treated both at birth and later in life (Walters, 1966; Nishizuka
et al., 1964; De Benedictis et al., 1962; Klein, 1966). On the contrary, the finding
of a high incidence of hepatomas only in males given NMUrea at birth in an
experiment lasting 60 weeks is similar to the observations following 20-methyl-
cholanthrene or urethane (Klein, 1959; Chieco-Bianchi et al., 1965; Klein, 1966).

Tumours of the forestomach were more numerous among mice given NMUrea
when mature. This result contrasts with the finding that carcinogenesis in
the forestomach by intragastric administration of 20-methylcholanthrene or
urethane was similar in mice treated as infants or later in life (Klein, 1959, 1966).

In rats, the higher incidence of anaplastic renal tumours among animals
treated at birth probably reflects a different susceptibility related to age. The
observation of stomach tumours only in rats given NMUrea at birth might indicate
a difference related to age at treatment, but the total number of tumours was
small; in any case, the present results confirm that NMUrea can induce stomach
tumours through a single parenteral administration (Druckrey et al., 1964). The
occurrence of some mammary tumours in female rats treated at birth or at 5 weeks
of age confirms a single previous observation (Kelly et al., 1968) and indicts the
mammary tissue of the female rat as another target organ for the carcinogenic
effect of NMUrea.

Among the tumours appearing at other sites in rats, and probably related
to the treatment, only one was neurogenic and was an intracranial neurinoma
(Table IV, Fig. 7). No tumours of this type were seen in the control rats. The
sporadicity of tumours of nervous tissue in rats and mice following a single i.p.
injection of NMUrea confirms a negative finding following a single intracranial
administration (Kelly et al., 1968) and contrasts with previous findings indicating

596

CARCINOGENICITY OF N-NITROSOMETHYLUREA                597

the nervous system as a major target for NMUrea. The latter results, however,
were obtained in experiments in which the carcinogen was given either intra-
venously (Druckrey et al., 1965; Fried and Fried, 1966; Janisch et al., 1967) or
orally with a long exposure (Stroobandt and Brucher, 1968; Thomas and Bollmann,
1969).

A common finding in the present series of experiments was a marked impair-
ment of body growth in mice and rats given NMUrea at birth. This effect was
unrelated to tumour development, since it was found also in tumour-free animals;
in addition, stunting growth was already obvious before weaning. Mice also
showed some hyaline changes in renal glomeruli, as previously described (Terracini
and Stramignoni, 1967). Other symptoms of homologous disease (Keast, 1968)
such as diarrhoea and hair loss were absent. The effect upon body growth in
animals treated at 5 weeks of age was much less marked or debatable.

The carcinogenicity of a single administration of NMUrea appears to be a
valuable tool for the study of dose-response relationships in carcinogenesis in
view of the effectiveness of the treatment and the rapid breakdown of the
carcinogen. Studies along this line are in progress in this laboratory.

The present investigation was supported by a grant from the Jane Coffin
Childs Memorial Fund for Medical Research. We are grateful to Mr. Gianfranco
Piovesana for technical help. The suggestions and the interest of Dr. G. Della
Porta are acknowledged.

REFERENCES

CHIECO-BIANCHI, L., FIORE-DONATI, L., TRIDENTE, G. and DE BENEDICTIS, G.-(1965)

Tumori, 51, 53.

DE BENEDICTIS, G., CHIECO-BIANCHI, L., TRIDENTE, G. AND FIORE-DONATI, L.-(1964)

Boll. Soc. ital. Biol. sper., 40, 610.

DE BENEDICTIS, G., MAIORANO, G., CHIECO-BIANCHI, L. AND FIORE-DONATI, L.-(1962)

Br. J. Cancer, 16, 686.

DELLA PORTA, G., CAPITANO, J., PARMI, L. AND COLNAGHI, M. I.-(1967) Tumori, 53, 81.
DELLA PORTA, G., COLNAGHI, M. I. AND PARMIANI, G.-(1968) Fd Cosmet. Toxic., 6, 707.
DELLA PORTA, G. AND TERRACINI, B.-(1969) Prog. exp. Tumor Res., 11, 334.

DOELL, R. G., DE VAUX ST. CYR, C. AND GRABAR, P.-(1967) Int. J. Cancer, 2, 103.

DOMSKY, I. I., LIJINSKY, W., SPENCER, K. AND SHUBIK, P.-(1963) Proc. Soc. exp. Biol.

Med., 113, 110.

DRUCKREY, H., IVANKOVIC, S. AND PREUSSMANN, R.-(1965) Z. Krebsforsch., 66, 389.

DRUCKREY, H., PREUSSMANN, R., IVANKOVIC, S. AND SCHMXHL, D.-(1967) Z. Krebs-

forsch., 69, 103.

DRUCKREY, H., STEINHOFF, D., PREUSSMANN, R. AND IVANKOVIC, S.-(1964) Z. Krebs-

forsch., 66, 1.

FREI, J. V.-(1969) Proc. Am. Ass. Cancer Res., 10, 26.

FRIED, R. AND FRIED, L. W.-(1966) Fedn Proc. Fedn Am. Socs exp. Biol., 25, 734.
GRAFFI, A. AND HOFFMANN, F.-(1966) Acta biol. med. germ., 17, 33.

JANISCH, W., SCHREIBER, D., STENGEL, R. AND STEFFEN, V.-(1967) Expl Path., 1, 243.
KEAST, D.-(1968) Adv. Cancer Res., 11, 43.

KELLY, M. G., O'GARA, R. W., YANCEY, S. T. AND BOTKIN, S.-(1968) J. natn. Cancer

Inst., 41, 619.

KLEIN, M.-(1959) Cancer Res., 19, 1109-(1966) J. natn. Cancer Inst., 36, 1111.
LEAVER, D. D., SWANN, P. F. AND MAGEE, P. N.-(1969) Br. J. Cancer, 23, 177.
MAGEE, P. N. AND BARNES, J. M.-(1962) J. Path. Bact., 84, 19.

598                 B. TERRACINI AND MARIA C. TESTA

MIRVISH, S. S., CIVIDALLI, G. AND BERENBLUM, I.-(1964) Proc. Soc. exp. Biol. Med.,

116,265.

NISHIZUKA, Y., NAKAKUKI, K. AND SAKAKURA, T.-(1964) Gann, 55, 495.
RIOPELLE, J. L. AND JASMIN, G.-(1969) J. natn. Cancer Inst., 42, 643.
STROOBANDT, G. AND BRUCHR, J. M.-(1968) Neurochirurgia, 14, 515.
TERRACINI, B. AND MAGEE, P. N.-(1964) Nature, Lond., 202, 502.

TERRACINI, B., PALESTRO, G., GIGLIARDI, M. R. AND MONTESANO, R.-(1966) Br. J.

Cancer, 20, 871.

TERRACINI, B. AND STRAMIGNONI, A.-(1967) Eur. J. Cancer, 3, 435.
THOMAS, C. AND BOLLMANN, R.-(1969) Experientia, 25, 50.
TOTH, B.-(1968) Cancer Res., 28, 727.

TOTH, B., RAPPAPORT, H. AND SHUBK, P.-(1963) J. natn. Cancer Inst., 30, 723.
WALTERS, M. A.-(1966) Br. J. Cancer, 20, 148.

				


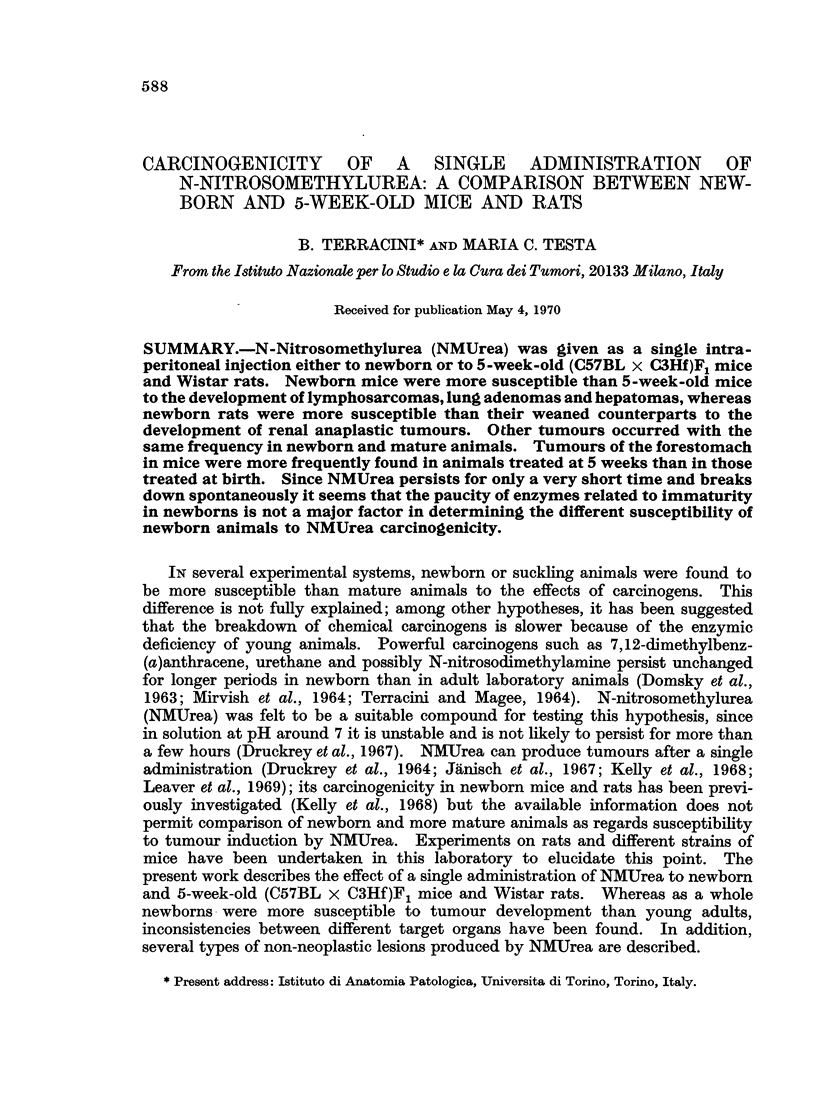

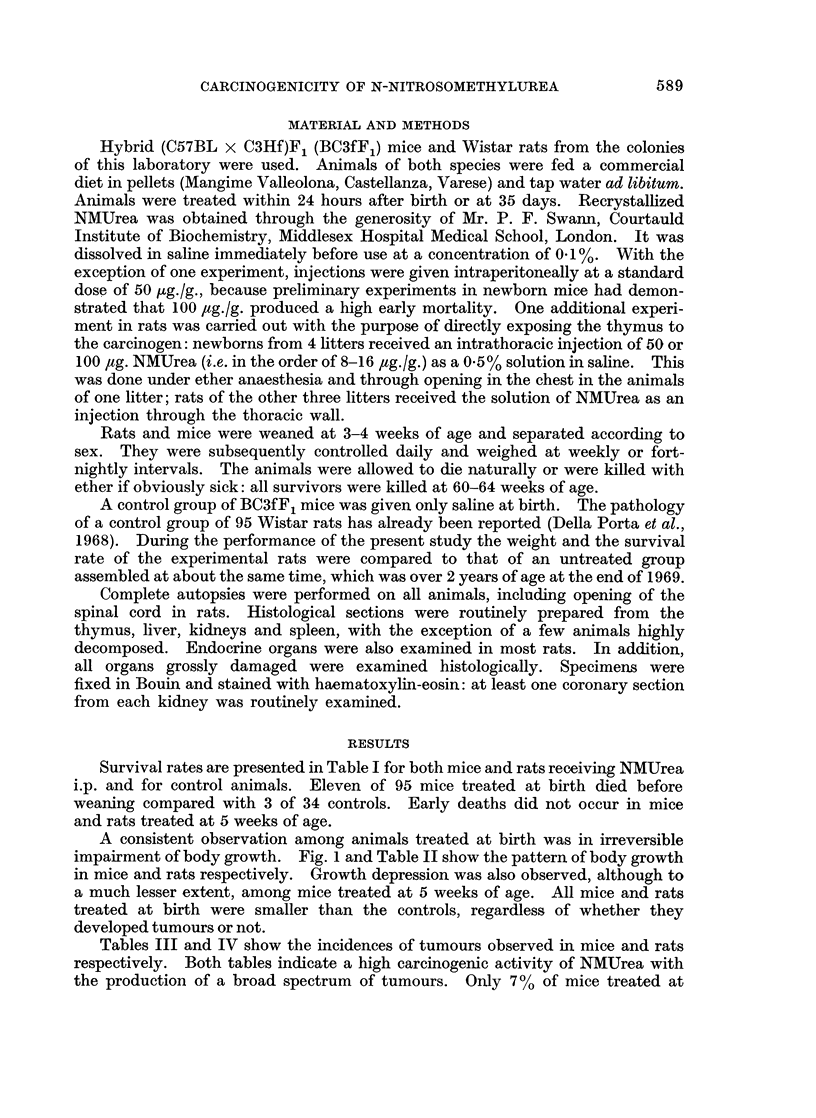

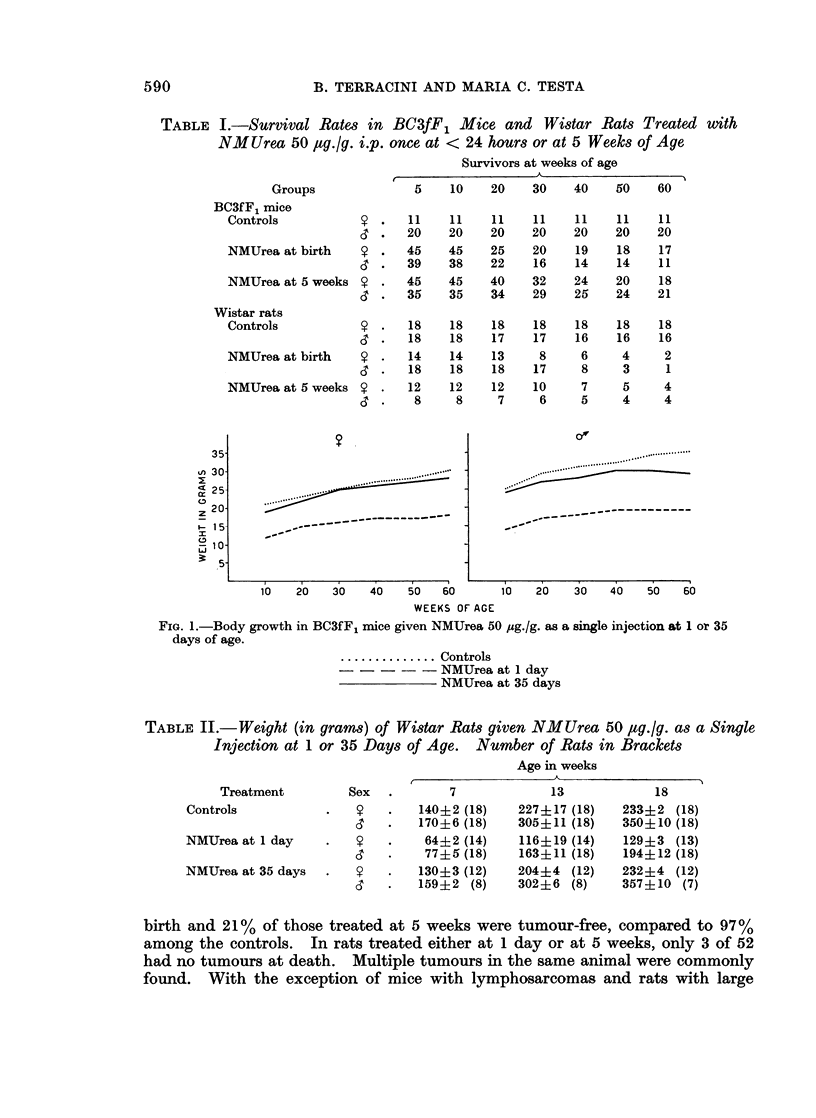

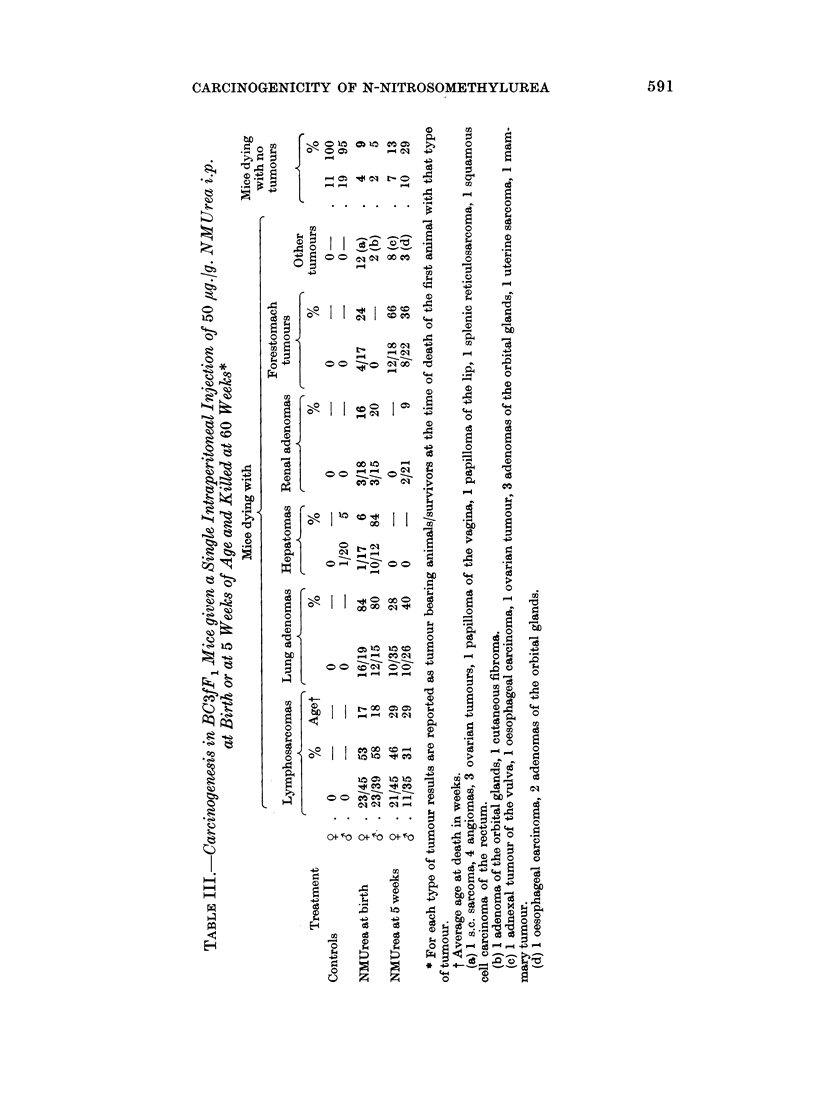

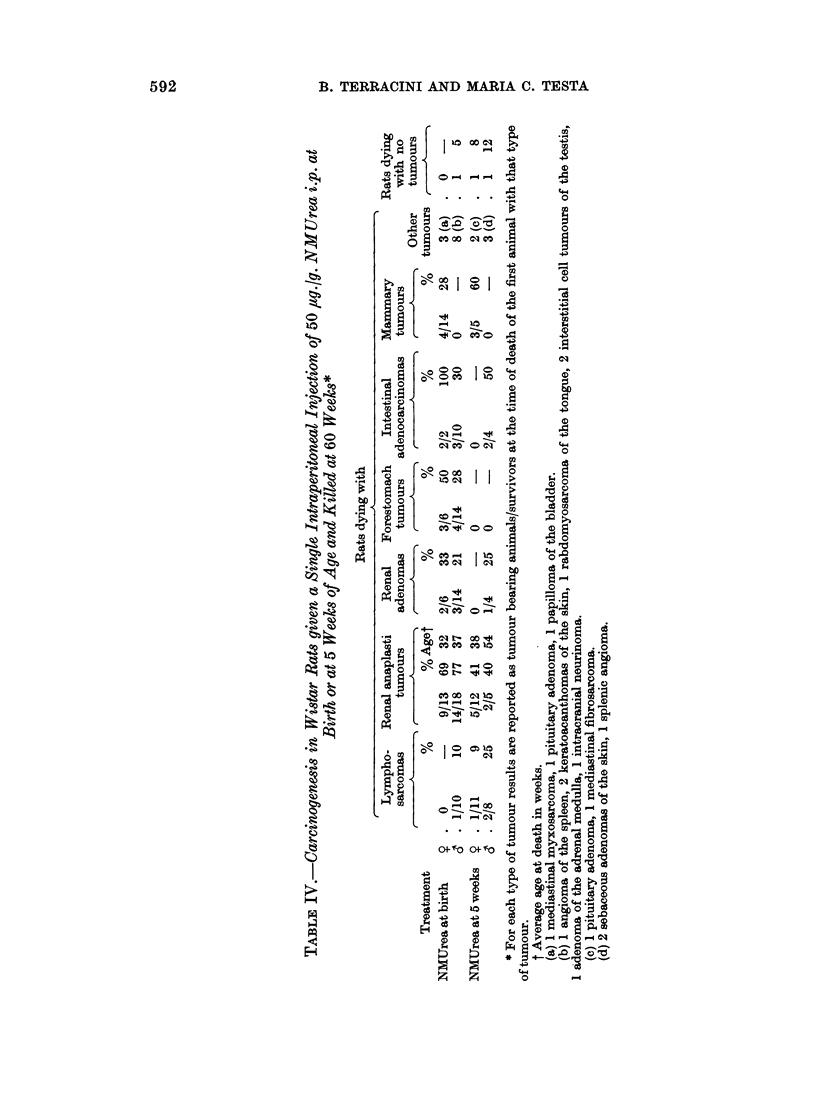

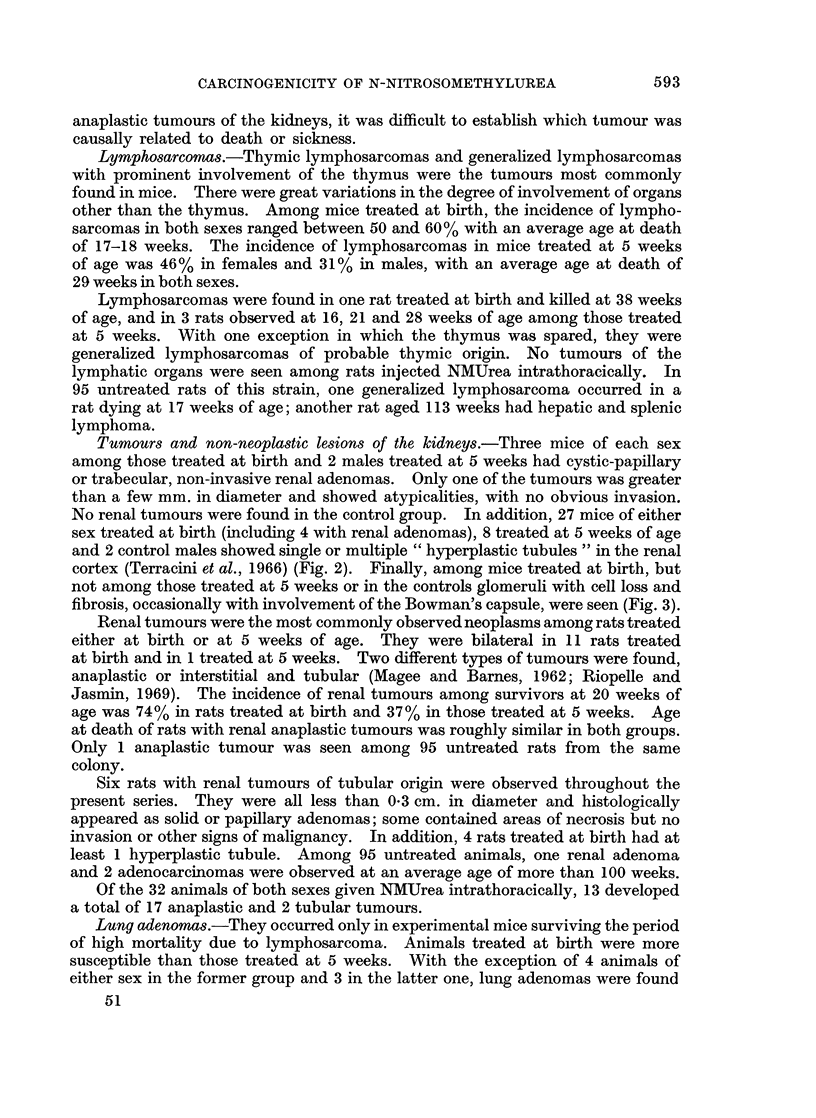

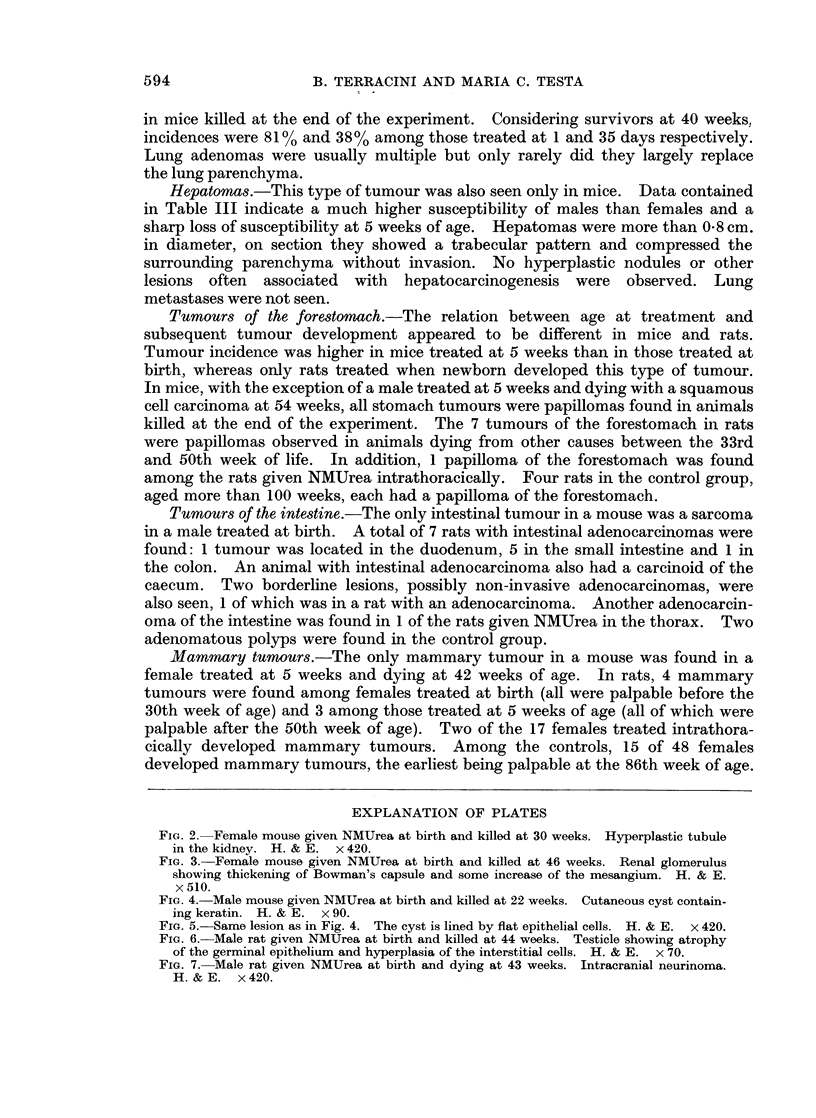

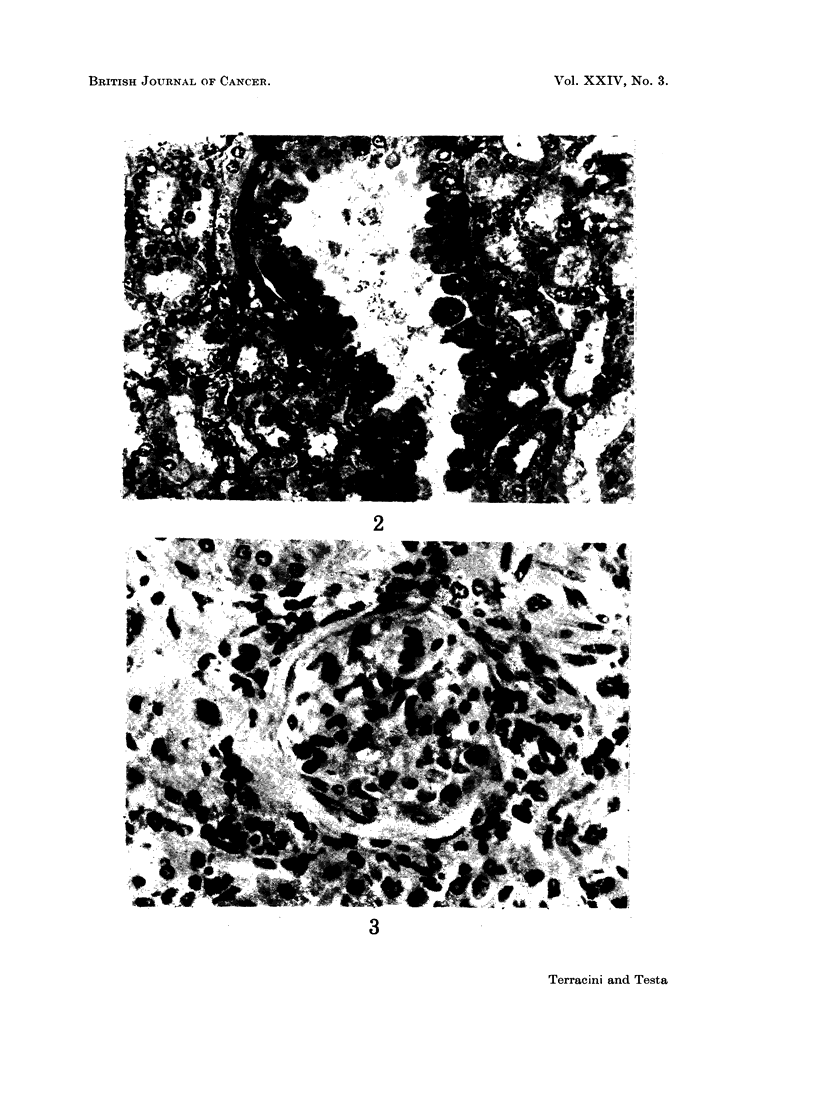

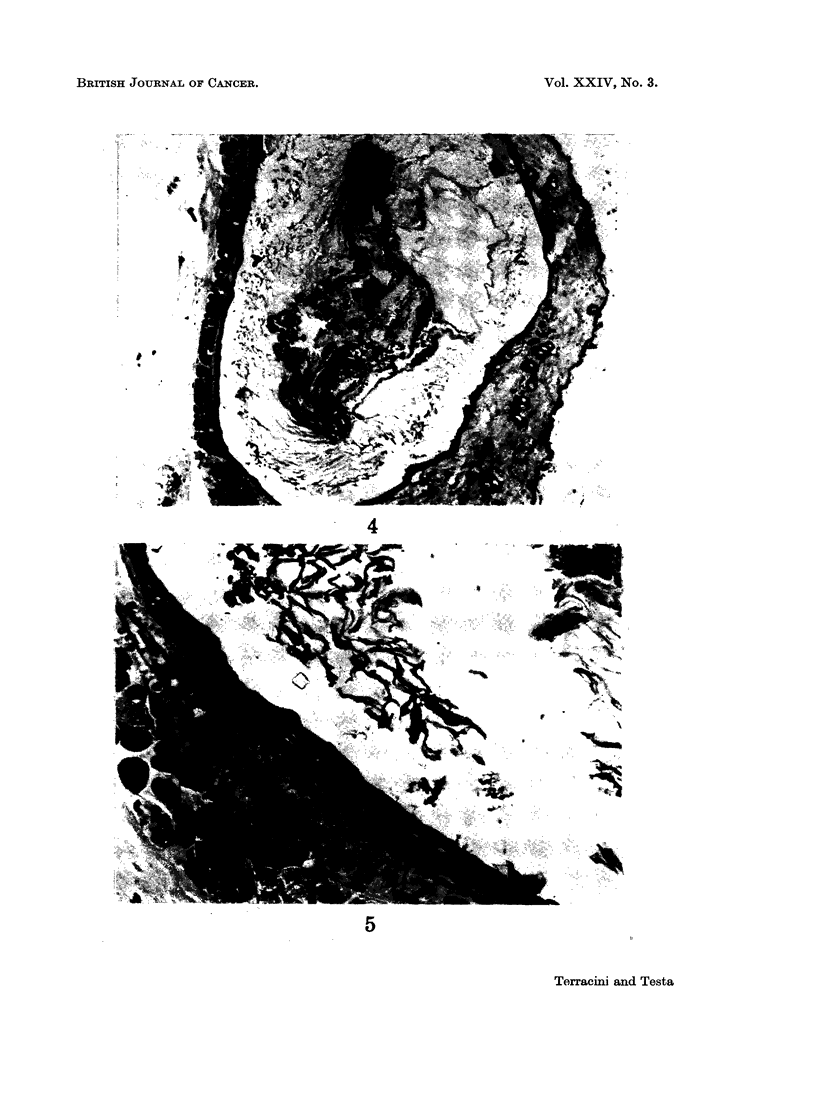

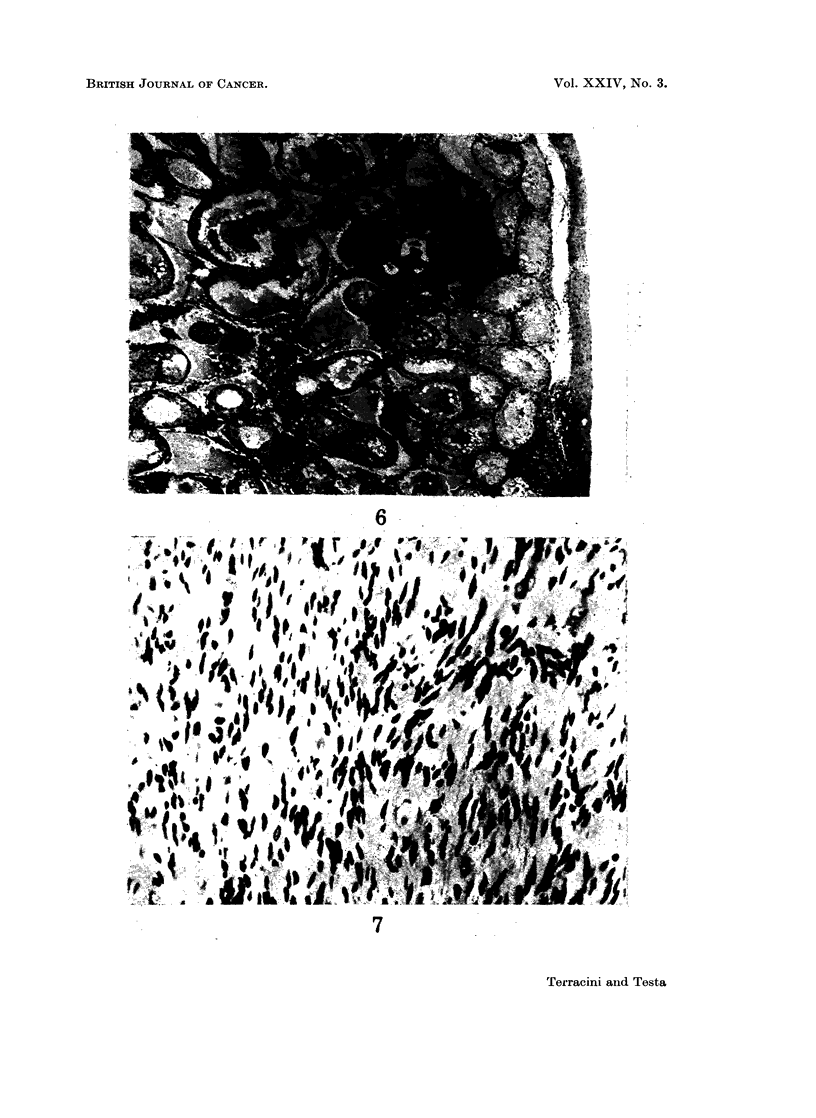

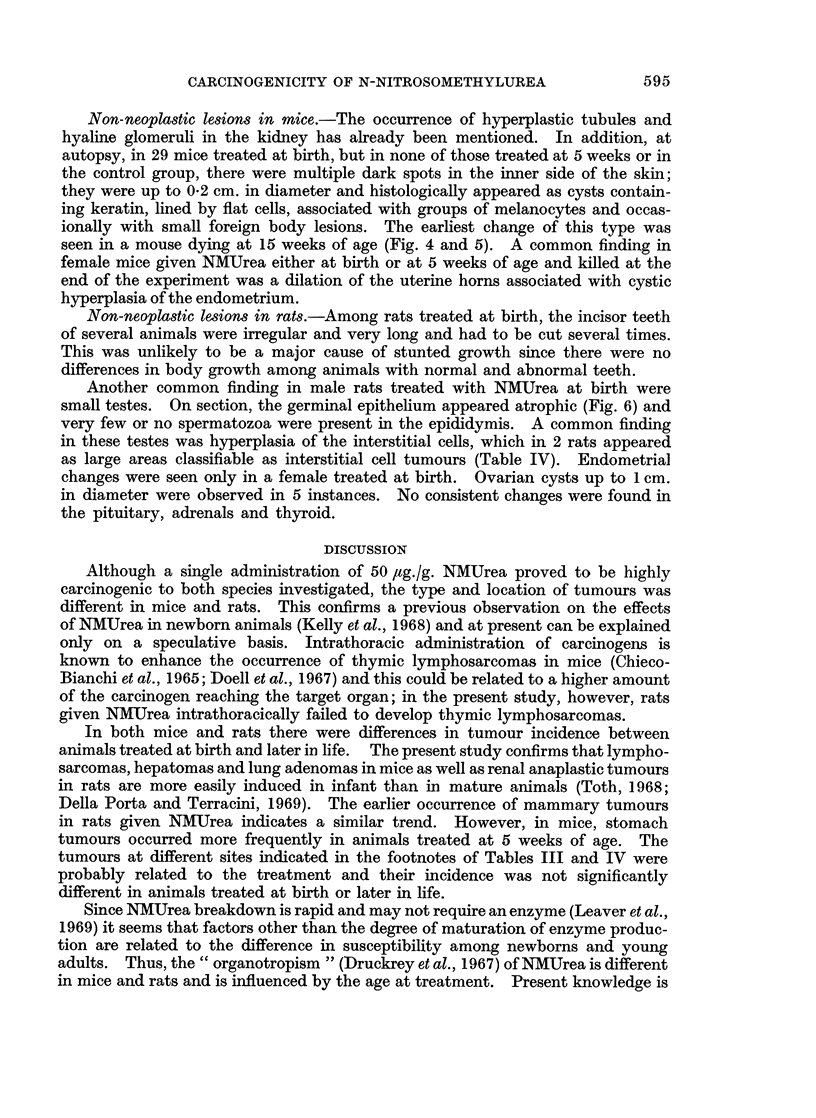

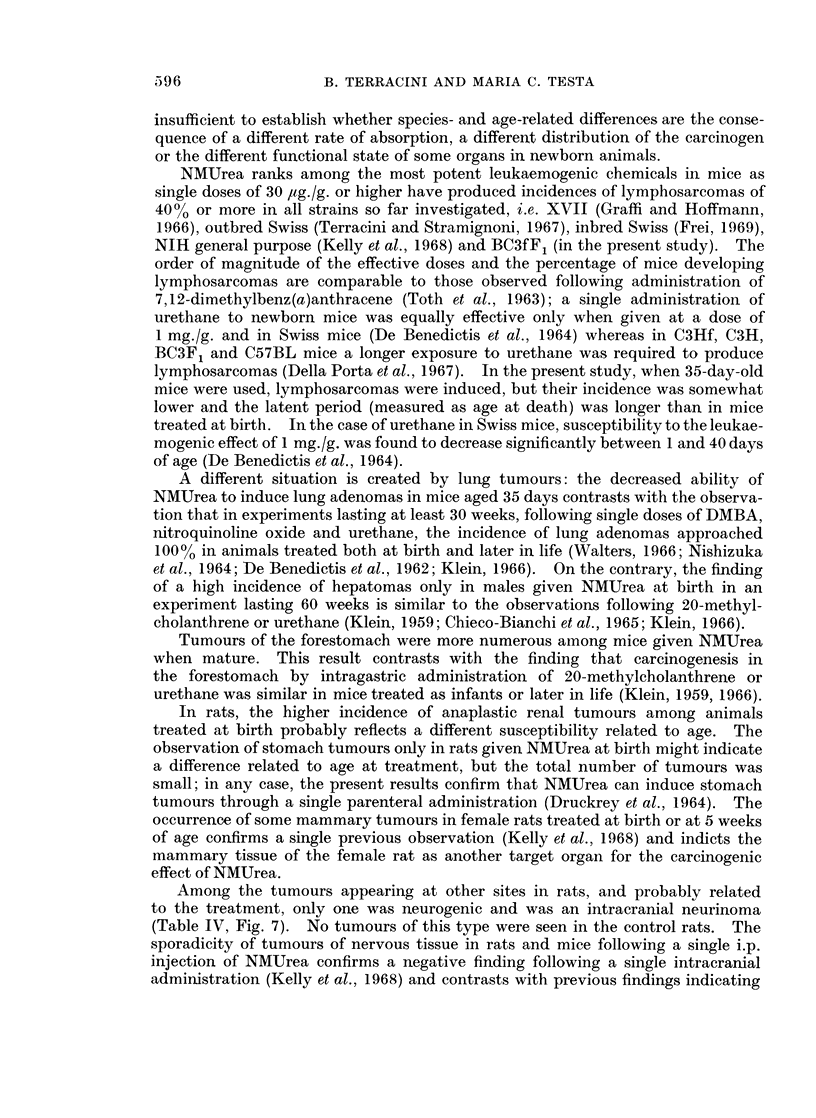

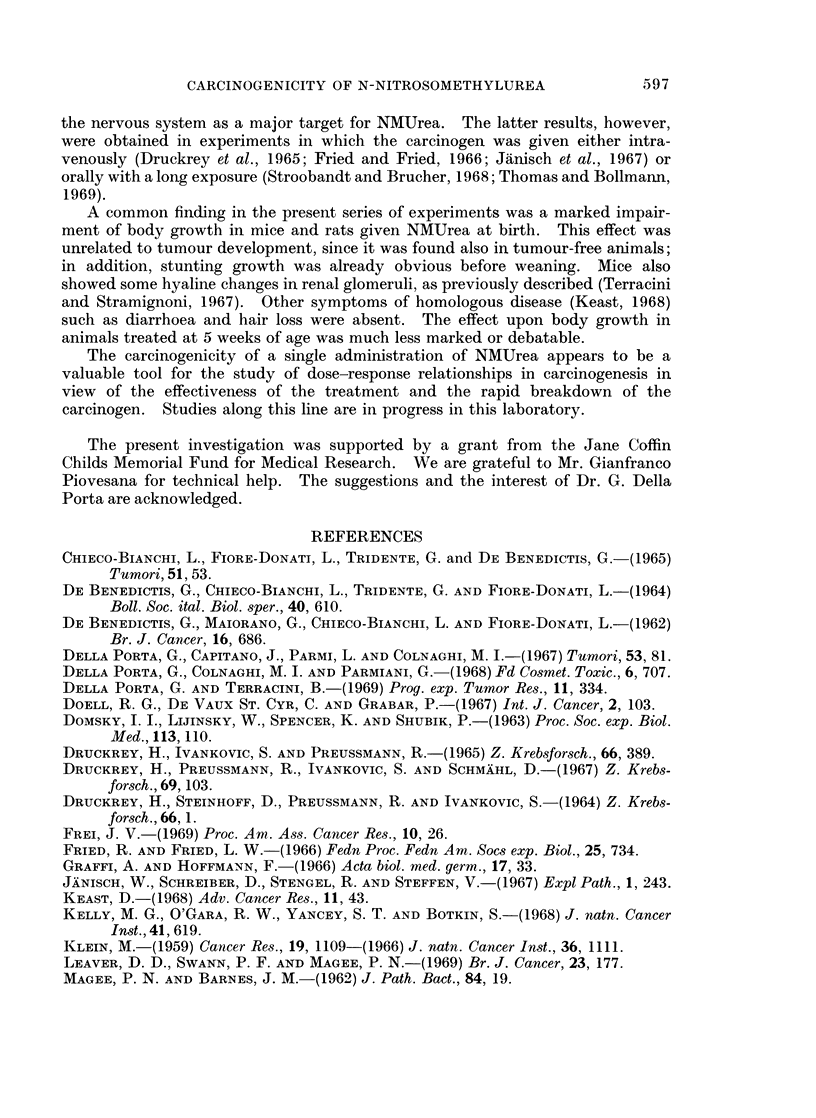

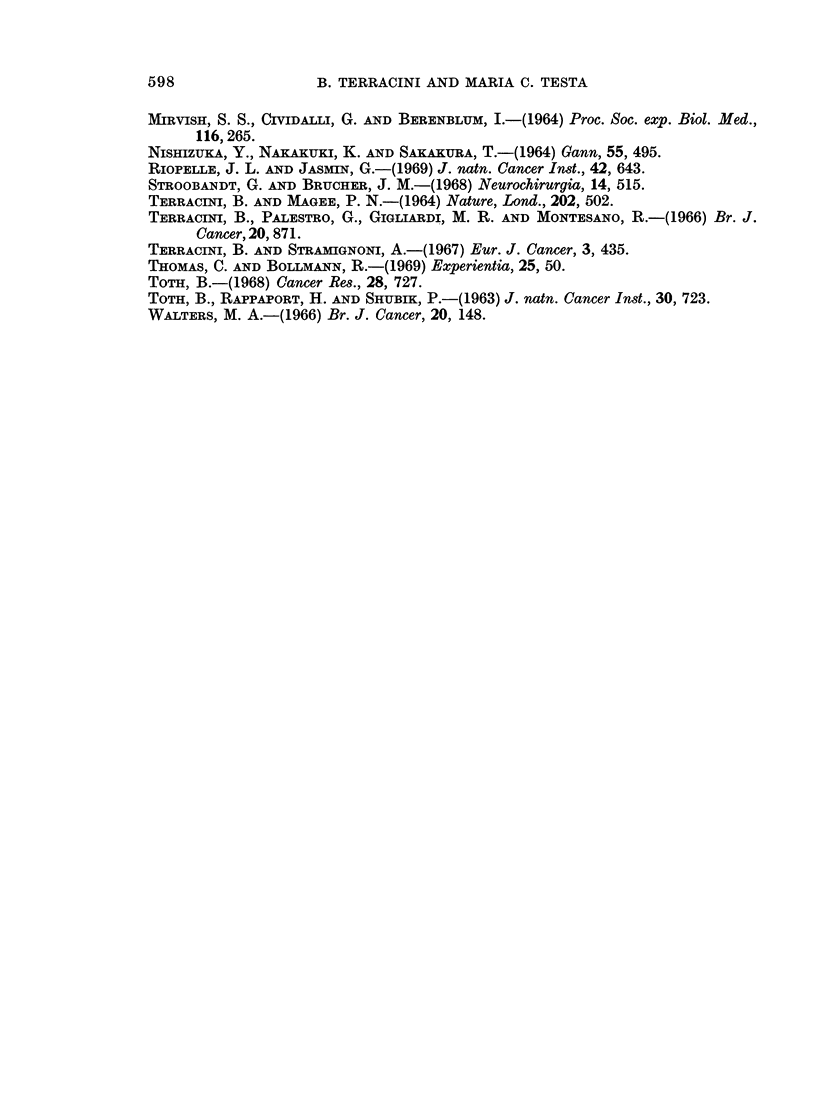


## References

[OCR_00970] DE BENEDICTIS G., MAIORANO G., CHIECO-BIANCHI L., FIORE-DONATI L. (1962). Lung carcinogenesis by urethane in newborn, suckling, and adult Swiss mice.. Br J Cancer.

[OCR_00980] DOMSKY I. I., LIJINSKY W., SPENCER K., SHUBIK P. (1963). Rate of metabolism of 9,10-dimethyl-1,2-benzanthracene in newborn and adult mice.. Proc Soc Exp Biol Med.

[OCR_00966] De Benedictis G., Chieco-Bianchi L., Tridente G., Fiore-Donati L. (1964). Influenza del fattore età sullo sviluppo di linfomi maligni e di epatomi in topi Swiss trattati con uretano (etil carbamato). Boll Soc Ital Biol Sper.

[OCR_00974] Della Porta G., Colnaghi M. I., Parmiani G. (1968). Non-carcinogenicity of hexamethylenetetramine in mice and rats.. Food Cosmet Toxicol.

[OCR_00976] Della Porta G., Terracini B. (1969). Chemical carcinogenesis in infant animals.. Prog Exp Tumor Res.

[OCR_00978] Doell R. G., De Vaux St Cyr C., Grabar P. (1967). Immune reactivity prior to development of thymic lymphoma in C57BL mice.. Int J Cancer.

[OCR_00986] Druckrey H., Preussmann R., Ivankovic S., Schmähl D. (1967). Organotrope carcinogene Wirkungen bei 65 verschiedenen N-Nitroso-Verbindungen an BD-Ratten.. Z Krebsforsch.

[OCR_01004] Kelly M. G., O'Gara R. W., Yancey S. T., Botkin C. (1968). Carcinogenicity of 1-methyl-1-nitrosourea in newborn mice and rats.. J Natl Cancer Inst.

[OCR_01007] Leaver D. D., Swann P. F., Magee P. N. (1969). The induction of tumours in the rat by a single oral dose of N-nitrosomethylurea.. Br J Cancer.

[OCR_01014] MIRVISH S., CIVIDALLI G., BERENBLUM I. (1964). SLOW ELIMINATION OF URETHAN IN RELATION TO ITS HIGH CARCINOGENICITY IN NEWBORN MICE.. Proc Soc Exp Biol Med.

[OCR_01016] NISHIZUKA Y., NAKAKUKI K., SAKAKURA T. (1964). INDUCTION OF PULMONARY TUMOR AND LEUKEMIA BY A SINGLE INJECTION OF 4-NITROQUINOLINE 1-OXIDE TO NEWBORN AND INFANT MICE.. Gan.

[OCR_01017] Riopelle J. L., Jasmin G. (1969). Nature, classification, and nomenclature of kidney tumors induced in the rat by dimethylnitrosamine.. J Natl Cancer Inst.

[OCR_01019] TERRACINI B., MAGEE P. N. (1964). RENAL TUMOURS IN RATS FOLLOWING INJECTION OF DIMETHYLNITROSAMINE AT BIRTH.. Nature.

[OCR_01021] Terracini B., Palestro G., Gigliardi M. R., Montesano G., Montesano R. (1966). Carcinogenicity of dimethylnitrosamine in Swiss mice.. Br J Cancer.

[OCR_01030] Walters M. A. (1966). The induction of lung tumours by the injection of 9,10-dimethyl-1,2-benzanthracene (DMBA) into newborn suckling and young adult mice. A dose response study.. Br J Cancer.

